# Nephroprotective Effect of Cilastatin against Gentamicin-Induced Renal Injury In Vitro and In Vivo without Altering Its Bactericidal Efficiency

**DOI:** 10.3390/antiox9090821

**Published:** 2020-09-03

**Authors:** Juan Carlos Jado, Blanca Humanes, María Ángeles González-Nicolás, Sonia Camaño, José Manuel Lara, Beatriz López, Emilia Cercenado, Julio García-Bordas, Alberto Tejedor, Alberto Lázaro

**Affiliations:** 1 Renal Physiopathology Laboratory, Department of Nephrology, Instituto de Investigación Sanitaria Gregorio Marañón (IiSGM), Hospital General Universitario Gregorio Marañón, 28007 Madrid, Spain; jadete84@hotmail.com (J.C.J.); blancahumanes@gmail.com (B.H.); rengac@yahoo.es (M.Á.G.-N.); csonia366@gmail.com (S.C.); atejedor@senefro.org (A.T.); 2 Department of Medicine, School of Medicine, Universidad Complutense, 28040 Madrid, Spain; 3 Department of Pathology, Hospital General Universitario Gregorio Marañón, 28007 Madrid, Spain; jmihq@yahoo.es (J.M.L.); bealopezmb@gmail.com (B.L.); julgarbor@hotmail.com (J.G.-B.); 4 Department of Microbiology, Hospital General Universitario Gregorio Marañón, 28007 Madrid, Spain; emilia.cercenado@salud.madrid.org; 5 Department of Physiology, School of Medicine, Universidad Complutense, 28040 Madrid, Spain

**Keywords:** cilastatin, acute kidney injury, nephrotoxicity, nephroprotection, apoptosis, oxidative stress, inflammation, megalin

## Abstract

Gentamicin is a used antibiotic that causes nephrotoxicity in 10–20% of treatment periods, which limits its use considerably. Our results have shown that cilastatin may be a promising therapeutic alternative in toxin-induced acute kidney injury (AKI). Here, we investigated its potential use as a nephroprotector against gentamicin-induced AKI in vitro and in vivo. Porcine renal cells and rats were treated with gentamicin and/or cilastatin. In vivo nephrotoxicity was analyzed by measuring biochemical markers and renal morphology. Different apoptotic, oxidative and inflammatory parameters were studied at cellular and systemic levels. Megalin, mainly responsible for the entry of gentamicin into the cells, was also analyzed. Results show that cilastatin protects cells from gentamicin-induced AKI. Cilastatin decreased creatinine, BUN, kidney injury molecule-1 (KIM-1) and severe morphological changes previously increased by gentamicin in rats. The interference of cilastatin with lipid rafts cycling leads to decreased expression of megalin, and therefore gentamicin uptake and myeloid bodies, resulting in a decrease of apoptotic, oxidative and inflammatory events. Moreover, cilastatin did not prevent bacterial death by gentamicin. Cilastatin reduced gentamicin-induced AKI by preventing key steps in the amplification of the damage, which is associated to the disruption of megalin-gentamicin endocytosis. Therefore, cilastatin might represent a novel therapeutic tool in the prevention and treatment of gentamicin-induced AKI in the clinical setting.

## 1. Introduction

Aminoglycoside antibiotics are among the most widely used drugs to treat Gram-negative bacterial infections worldwide due to their high efficiency, and gentamicin is probably the most commonly used aminoglycoside [1,2]. Nevertheless, its use has been compromised owing to nephrotoxic side effects in 10–20% of therapeutic regimens [3]; this percentage reaches 50% after 14 days of therapy [4]. Renal toxicity is therefore a clinical problem resulting in increased morbidity during and after gentamicin therapy and leading to acute kidney injury (AKI) [2,5].

Although the mechanisms underlying gentamicin-induced AKI are not fully understood, it is known that renal damage is directly related to the transport and accumulation of the drug in the renal proximal tubular epithelial cells (RPTECs). Several studies have pointed to megalin, a multiligand endocytic receptor located in the apical brush border of the RPTECs, as the entity responsible for the uptake of gentamicin and, therefore, its accumulation in the kidneys [5,6,7]. After binding to megalin, gentamicin is internalized via endocytosis and transported to the Golgi bodies, endoplasmic reticulum and lysosomes, where it destabilizes lipid metabolism with the accumulation of phospholipids, reduced phospholipase activity and finally phospholipidosis, which is closely related to gentamicin-induced nephrotoxicity and cell death [2,8,9]. Release of gentamicin (together with calpains and cathepsins) into cytoplasm after the rupture of membranes activates proapoptotic proteins that directly damage the mitochondria, thus triggering the pathways of apoptosis, the production of reactive oxygen species (ROS) and an inflammatory response [10]. Together, these finding contribute to the development and amplification of renal damage [2,8,10,11].

Currently, up to 10% of all cases of AKI are attributed to gentamicin and consequently, it is essential to find alternative therapies to prevent the undesired renal side effects of gentamicin [12].

Based on previous studies with other nephrotoxic drugs, we show that inhibition of the renal dehydropeptidase-I (DHP-I) enzyme located in the cholesterol lipid rafts (CLRs) on the brush border apical side of RPTECs can protect against AKI induced by these agents regardless of their chemical nature or physical properties. In fact, we specifically demonstrated that the use of cilastatin, a dipeptidyl analog that reversibly binds to DHP-I, was able to inhibit cyclosporine-, tacrolimus-, vancomycin- and cisplatin-induced apoptosis in RPTECs. In addition to protecting against apoptosis in animal studies, cilastatin also protected against oxidative and inflammatory damage, with no reduction in the drug’s therapeutic effects on their target cells [13,14,15,16,17,18].

Thus, the present study aims to examine the potential beneficial effects of cilastatin against gentamicin-induced renal injury in vitro and in vivo, as well as to investigate the main underlying pathogenic mechanisms. We hypothesized that cilastatin improves renal injury by preventing oxidative stress (OS) and an inflammatory response related to the blockade of the intrinsic and extrinsic pathways of the apoptosis in gentamicin-induced AKI without compromising the drug’s inherent bactericidal properties.

## 2. Materials and Methods

### 2.1. Drugs

Gentamicin sulphate and crystalline cilastatin were obtained from Acofarma^®^ (Barcelona, Spain) and Merck Sharp and Dohme S.A. (Madrid, Spain) respectively. Both gentamicin and cilastatin were dissolved in a cell culture medium at the specified concentrations for the in vitro studies and in normal 0.9% saline solution for the in vivo studies.

### 2.2. Experimental Animals

Miniature swine aged 3 months, weighing 31.3 kg on average and being either male or female (Agricultural Complex, Technological Institute for Agricultural Development, Aranjuez, Community of Madrid, Spain) were used to obtain the primary cultures of RPTECs.

In vivo studies were performed on male Wistar rats (raised in the IiSGM animal facility) aged 7–8 weeks and weighing 284 g, on average. They were weighed every day and housed under light (12 h light–dark cycle) in a temperature- and humidity-controlled environment with free access to food and water.

The studies were started after approval by the Ethics Committee for Animal Experimentation of Gregorio Marañón Hospital (GMH, Madrid, Spain). Animals were handled in accordance with Directive 2010/63/EU and Spanish Royal Decree 53/2013 on the protection of animals used for experimentation and other scientific purposes. The registration code of the Ethics Committee in Animal Experimentation for this project granted by the Gregorio Marañon Institute for Health Research is 15/2011.

### 2.3. Renal Proximal Tubular Primary Cell Culture

Porcine RPTECs were obtained as previously reported [13,14,15]. Briefly, a microtome Staddie Riggs (Thomas Scientific, US) was used to prepare thin slices from the renal cortex followed by 30 min incubation in Ham’s F-12 medium at 37 °C with 0.6 mg/mL of collagenase A (Boehringer Mannheim, Germany). Digested tissue was filtered out using a metal mesh (250 µm), washed repeatedly (3×) with Ham’s F-12 medium and centrifuged with an isotonic Percoll gradient (45% (*v*/*v*); 20,000× *g*; 30 min). Proximal tubules located in the deepest fraction after centrifugation were collected, washed again 3 times and resuspended in supplemented Dulbecco’s modified Eagle’s medium/Ham’s F-12 medium at a 1:1 ratio (with 3.7 mg/mL sodium bicarbonate, 25 mM HEPES, 1% non-essential amino acids, 2.5 mM glutamine, 100 U/mL penicillin, 100 mg/mL streptomycin, 5 mg/mL insulin-transferrin-sodium selenite media supplement, 5 × 10^−8^ M hydrocortisone and 2% fetal bovine serum). Proximal tubules were finally seeded at a density of 0.66 mg/mL and incubated at 37 °C in a 95% air/5% CO2 atmosphere. The cell medium was replaced every 48 h, and cells were ready for the experiment once they reached 80–85% confluence.

### 2.4. Cell Death Study in RPTECs

In all the assays RPTECs were treated with 10, 20 and 30 mg/mL of gentamicin in the presence or absence of cilastatin (200 µg/mL) for 24 h. In this work, a single dose of cilastatin was used, which has been shown to be protective against cyclosporine and cisplatin induced cell death [13,14]. We have previously demonstrated that higher doses of cilastatin (tested range between 50 and 1000 µg/mL) exert greater protection against toxicants (dose–response effect) but without affecting the normal growth of RPTECs [13]. The selection of 200 µg/mL was therefore made because it is the highest approximate concentration of cilastatin reached in plasma for the range of doses of imipenem-cilastatin used in a human clinic [19,20].

#### 2.4.1. Morphology Analysis

Images of cell morphology were obtained with an Olympus IX70 inverted microscope (Olympus, Hamburg, Germany) in the phase-contrast mode.

#### 2.4.2. Quantification of Cell Detachment

Detached cells were evaluated using flow cytometry (Gallios Beckman Coulter, Barcelona, Spain), as previously described [15]. In brief, after treatment of RPTECs with different concentrations of gentamicin with or without cilastatin, the floating cells were collected from the supernatant, resuspended in 300 µL of PBS and quantified in the cytometer. The samples were passed at a mean flow rate of 60 μL/min for 180 s in all cases, obtaining results such as cell count vs. size (FS; forward scatter) and complexity (SS; side scatter). The Kaluza analysis software (Beckman Coulter) was used to analyze the data.

#### 2.4.3. Localization of Cleaved Caspase 3

RPTECs were grown on sterile glass coverslips and treated with gentamicin with or without cilastatin for 24 h. Cells were incubated with antibody against cleaved caspase-3 (Asp175; 1:50; Cell Signaling Technology, Inc., Beverly, MA, USA). Immunolocalization was examined with the 20× PL-APO 0.7 numerical aperture objective of a Leica-SP2 confocal microscope (Leica Microsystems, Heidelberg, Germany). The staining analysis was performed with Leica Confocal Software LCS-1537 (Leica Microsystems).

#### 2.4.4. Nucleosomal Quantification

DNA fragments were measured using the Cell Death Detection ELISAPLUS Kit (Boehringer-Mannheim, Roche, Germany) according to the manufacturer’s instructions. After the incubation time with gentamicin and/or cilastatin, RPTECs were lysed for 30 min at room temperature and centrifuged at 200× *g* for 10 min to remove cell debris. Of the resulting supernatant 20 µL was added to the ELISA plate, and DNA and histones were quantified by reading the absorbance at 405 nm in a microplate reader (Synergy HT, BioTek, Winooski, VT, USA).

### 2.5. In Vivo Model and Experimental Protocols

The study was based on 28 animals randomly distributed into 4 groups: untreated controls (*n* = 6); cilastatin-treated rats (*n* = 6); gentamicin-administered rats (*n* = 8) and cilastatin-treated gentamicin-administered rats (*n* = 8). Gentamicin 80 mg/kg or its vehicle was injected to the rats intraperitoneally (i.p.) once a day until the end of the study in the same conditions and volume (5 mL/kg). Cilastatin 150 mg/kg was also administered every 24 h by i.p. injections (0.5 mL/kg) from the first dose of gentamicin until euthanasia, and it was administered to animals immediately before gentamicin. In the groups that were not treated with cilastatin, it was replaced by its vehicle. The route of administration and dose used of gentamicin were based on a pilot study involving induction of nephrotoxicity in rats, as described elsewhere [3]; the route of administration and dose of cilastatin were based on and readjusted to previous experiences by our group, taking into account earlier evaluations showing that cilastatin was able to prevent cisplatin-induced AKI [16,17].

One day before euthanasia, the animals were housed in metabolic cages (Harvard Apparatus^®^, Ref, ST1 52-6707), the urine of each rat was collected, and the volume was measured over a 24 h period. On the ninth day, all rats were anesthetized with ketamine (10 mg/kg) and diazepam (4 mg/kg) and euthanized. Blood samples were immediately collected from the abdominal aorta, and serum was separated for biochemical analysis. The kidneys were perfused with cold saline solution and quickly removed and decapsulated. Kidney samples were snap-frozen in liquid nitrogen and kept at –80 °C or fixed in 4% paraformaldehyde and paraffin-embedded for the different studies.

### 2.6. Renal Function Parameters

Serum and urine creatinine, blood urea nitrogen (BUN) levels, urine creatinine and serum and other parameters were measured with the Dimension RxL autoanalyzer (Dade-Behring, Siemens, Germany). The glomerular filtration rate (GFR) was obtained by measuring the creatinine clearance rate. Furthermore, the sulfosalicylic method was considered to calculate proteinuria [16].

### 2.7. Renal Histology Studies

A hematoxylin and eosin stain (Sigma-Aldrich, St Louis, MO, USA) was performed in paraffin-embedded renal sections (4 µm). The kidney injury score was calculated as previously reported by two independent pathologists in a blinded fashion [16,17].

Electron microscopy was performed for 2 kidneys randomly selected from each study group. For this purpose, frozen kidney tissues were fixed for 24 h in 2% glutaraldehyde. They were then cut into 1 mm^3^ blocks and post-fixed for 24 h in 2% osmium tetroxide. After washing with PBS, tissue blocks were dehydrated in increasing grades of ethanol and embedded in an epoxy resin (Spurr). Thick sections (1 µm) were cut with glass knives on a Reichert-jung UltraCut E Ultramicrotome (Reichert Technologies, Depew, NY, USA), stained with toluidine blue and analyzed by optical microscopy to identify the tissue. Once the cutting area was selected, ultrathin sections (800 Å thick) were taken with a diamond knife, mounted on 300-mesh nickel grids (TAAB Laboratories Equipment Ltd., Berks, UK) and stained with 2% uranyl acetate for 30 min at 60°. After washing with distilled water, samples were stained with lead citrate for 20 min and then examined in a JEOL JEM-100 SX Electron Microscope (JEOL Ltd., Tokyo, Japan).

### 2.8. Western Blots and Immunohistochemistry

Both techniques were performed as previously described [16,17]. Primary antibodies for Western blots were as follows: rabbit anti-Bax polyclonal antibody, 1:500; rabbit anti-Bcl-2 polyclonal antibody, 1:200; goat anti-caspase-3 p17 polyclonal antibody, 1:200; rabbit anti-Fas Ligand (FasL) polyclonal antibody, 1:200; goat anti-catalase polyclonal antibody, 1:1000; goat anti-heat shock protein (HSP)-27 polyclonal antibody, 1:200 (Santa Cruz Biotechnology, Inc., Santa Cruz, CA, USA); rabbit anti-caspase-9 (rat specific) polyclonal antibody, 1:1000; rabbit anti-cleaved caspase-3 (Asp175) polyclonal antibody, 1:1000 (Cell Signalling Technology, Inc., Beverly, MA, USA); goat anti-kidney injury molecule-1 (KIM-1) polyclonal antibody, dilution 1:2000 (R&D Systems, Minneapolis, MN, USA; dilution); rabbit anti-Fas/Apo1 polyclonal antibody (1:300; BioVision Research Products, Mountain View, CA, USA); rabbit anti-Cu/Zn SOD polyclonal antibody, 1:4000 and mouse anti-HSP-70 monoclonal antibody, 1:1000 (Assay Designs, Stressgen, Ann Arbor, MI, USA). As an internal standard, membranes were reprobed with a mouse anti β-actin monoclonal antibody, 1:5000 (Sigma-Aldrich, St. Louis, MO, USA) to verify the equal loading of protein in each line. All signals were visualized with an Alliance 4.7 (Uvitec, Cambridge, UK) and analyzed by densitometric scanning using ImageJ [17]. Results were expressed in arbitrary units (a.u.).

The primary antibodies used for the immunohistochemical studies were: goat anti-KIM-1 polyclonal antibody (R&D Systems; dilution 1:20); rabbit anti-cleaved caspase-3 (Asp175) polyclonal antibody (Cell Signaling Technology, Inc.; dilution 1:50); rabbit anti-Cu/Zn SOD polyclonal antibody (Assay Designs, Stressgen, dilution 1:1000); mouse anti-4-hydroxy-2-nonenal (4-HNE) monoclonal antibody (Oxis International Inc., Foster City, CA, USA; dilution 1:75); mouse anti-CD 68 monoclonal antibody (AbD Serotec, Oxford, UK; dilution 1:125); goat anti-vascular cell adhesion protein-1 (VCAM-1) polyclonal antibody (Santa Cruz Biotechnology; dilution 1:100); goat anti-transforming growth factor beta 1 (TGFβ-1) polyclonal antibody (Santa Cruz Biotechnology; dilution 1:500); goat anti-connective tissue growth factor (CTGF) polyclonal antibody (Santa Cruz Biotechnology; dilution 1:1000) and rabbit anti-megalin polyclonal antibody (Santa Cruz Biotechnology; dilution 1:100). The specificity of the antibodies was verified by controls lacking the primary antibody, producing no background. The surface area labeled by anti-4-HNE, anti-VCAM-1, anti-TGFβ-1, anti-CTGF or anti-megalin antibodies was evaluated by quantitative image analysis as previously reported [17]. CD68 positive cells were counted by considering the number of total positive cells per mm^2^ of renal tissue.

### 2.9. TUNEL Assay

DNA fragmentation was measured in 4-µm kidney tissue sections using a Fluorescein FragEL DNA Fragmentation Detection kit (Calbiochem, CA, USA) following the manufacturer’s instructions. TUNEL results were examined with the Leica-SP2 confocal microscope (Leica Microsystems, Heidelberg, Germany). The staining analysis was performed by counting all the TUNEL-positive cells in four non-overlapping fields for each sample.

### 2.10. Measurement of TNFα and Antioxidant Capacity

Serum TNFα and urine total antioxidant capacity were measured using the Rat TNFα ELISA kit (Thermo Fisher Scientific, MA, USA) and Oxiselect Total Antioxidant Capacity Assay Kit (Cell Biolabs, CA, USA), respectively, according to the manufacturer’s protocol. For the TNFα measurement, 50 µL of serum was tested on the ELISA plate and its quantification was obtained by interpolating the difference of the absorptions at 450 nm and 550 nm in a standard curve, expressing the final result in pg/mL. The antioxidant capacity was measured by mixing the urine sample (20 µL) with 180 µL of the reaction buffer previously diluted in PBS 1X (1:100). The initial absorbance (490 nm) was read in order to calculate the net absorbance (final absorbance minus initial absorbance), and 50 µL of the copper ion reagent was added and then stirred for 5 min. The reaction was stopped by adding 50 μL of the stop solution and the final absorbance was read at 490 nm. The antioxidant capacity was calculated by comparing the net absorbance values obtained in duplicate with the standard curve. Values are shown in mM.

### 2.11. Determination of Gentamicin Levels

RPTECs incubated for 24 h with 10, 20 and 30 mg/mL of gentamicin in the absence or presence of cilastatin (200 µg/mL), were scraped and lysed in 400 µL of lysis buffer at 70 °C (19.33% (*v*/*v*) glycerol (87% *v*/*v*); 2.22% (*w*/*v*) SDS; 790 mM Tris-HCl pH 6.8 in dH_2_O, 0.1 M phenylmethylsulfonyl fluoride (PMSF) and 10 µL/mL protease inhibitors (Sigma)). Cell lysates were heated at 100 °C for 5 min, homogenized in ice and centrifuged at 12,000× *g* for 5 min at 4 °C.

Renal tissue was pulverized and digested with lysis buffer (2 mM EDTA, 150 mM NaCl, 50 mM Tris-HCl, 2 mM EGTA, 0.3% NP-40, 0.2% Triton X-100, 0.1 mM PMSF and 10 µL/mL protease inhibitor cocktail). Lysates were homogenized in ice with agitation for 20 min and centrifuged at 12,000 rpm for 15 min at 4 °C.

In both cases the supernatants were analyzed for the total protein content (by the BCA colorimetric method—Pierce™, Thermo Scientific™, Rockford, IL, USA) and gentamicin. Gentamicin was measured using a TDX FLx^®^ analyzer (Abbott Laboratories, Abbott Park, IL, USA) via fluorescence polarization immunoassay technology according to the manufacturer’s recommendations. Results were shown as µg gentamicin/g protein for the renal tissues and µg gentamicin/µg protein for RPTECs.

Gentamicin content in urine was determined by diluting the urine 1/250 in saline serum; a 100 µL diluted sample was analyzed in a clinical chemistry analyzer Viva/ProE System (Siemens, Erlangen, Germany) following the manufacturer’s protocol. Results were expressed as µg gentamicin/mL.

### 2.12. Megalin mRNA Expression

Total RNA extraction from the rat kidneys, reverse-transcription and real-time PCR were performed as previously described [17]. The primer sets used for megalin are forward-GACAACATCACTGCCCACAC and reverse-CACTCCAGAAGACACGACCA, and for GAPDH: forward-CGGCCGAGGGCCCACTAAAG and reverse-TGCTCAGTGTTGGGGGCTGAGT. These primer sequences were synthesized commercially (Invitrogen). Statistical analysis was carried out for at least six to eight experimental samples. The measurements were performed in duplicate. Results for double-distilled water controls were negative in the assays.

### 2.13. Bacterial Susceptibility Assays

Eight unique clinical isolates collected from patient body fluids were kindly provided by the Microbiology Department, GMH for the performance of the assays. The isolates correspond to 4 different *Staphylococcus aureus* strains and *Escherichia coli* strains.

Previous minimum inhibitory concentrations (MICs, concentration required to inhibit the visible growth of a microorganism after a 24 h period of incubation at 35 °C) and a microdilution testing-based assay (MicroScan panels, Siemens, Sacramento, CA, USA) confirmed that all tested bacteria were susceptible to gentamicin. To calculate MICs the broth microdilution method was performed with standard cation-adjusted Mueller–Hinton broth as previously specified by the Clinical and Laboratory Standards Institute guidelines [15]. Gentamicin was tested at dilutions ranging (1:2 serial dilutions) from 64 to 0.0625 µg/mL with or without cilastatin (200 μg/mL). To determine the minimum bactericidal concentrations (MBCs, concentration of the drug required to kill 99.9% of the microorganisms after a 24 h incubation period), 0.1 mL from the MIC well was taken and 4 more dilutions were cultured in blood agar plates and incubated for 24–48 h at 37 °C [14]. The MBCs values were recorded as the lowest dilution decreasing ≥99.9 in growth (≥3-log 10 reduction in colony-forming units (CFU)/mL) compared with the control. The results obtained with gentamicin in the presence or absence of cilastatin were analyzed and compared.

### 2.14. Statistical Methods

The statistical analysis was carried out using SPSS 11.5 (SPSS, Chicago, IL, USA). Quantitative variables were summarized as the mean ± standard error of the mean (SEM). Equality of variances was assessed using the Levene’s test. Normally distributed continuous variables were analyzed using the analysis of variance (ANOVA), with the least significant difference test as a post hoc approach to determine specific group differences. Data on urinary gentamicin excretion were analyzed using the *t*-test. Differences were considered statistically significant if bilateral α values were *p* < 0.05.

## 3. Results

### 3.1. Cilastatin Prevents Apoptosis and Reduces the Accumulation of the Antibiotic in Gentamicin-Treated RPTECs

**Figure 1:**
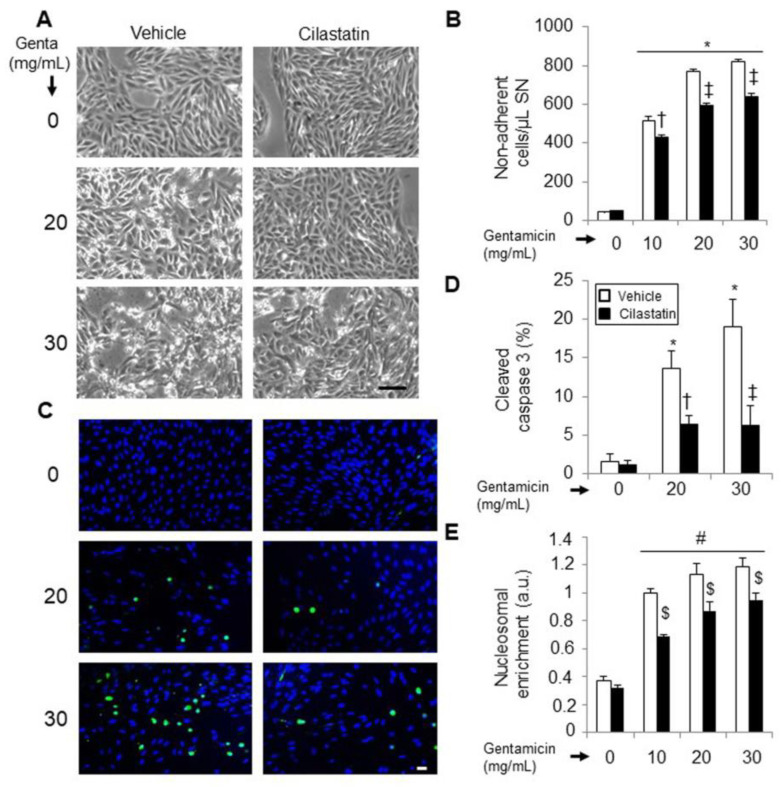
Effects of cilastatin on gentamicin-induced cytotoxicity and apoptosis in renal proximal tubular epithelial cells (RPTECs). RPTECs were administered with gentamicin (10, 20 or 30 mg/mL) in the presence and absence of cilastatin (200 µg/mL) for 24 h. (**A**) Phase-contrast photomicrographs (representative example of at least three independent experiments; original magnification, 40×, Bar, 100 µm); (**B**) effect of cilastatin on the gentamicin-induced detachment of RPTECs was measured by flow cytometry and determined by counting the number of cells in an equal volume of buffer and time and (**C**) confocal microscopy images of immunofluorescence localization of cleaved caspase-3. Green fluorescent staining indicates cleaved caspase-3 and blue staining (4, 6-diamidino-2-phenylindole (DAPI)) represents all nuclei in the sample (magnification, 20×, Bar, 25 µm); (**D**) quantification of cleaved caspase-3 fluorescence intensity and (**E**) oligonucleosomal DNA fragmentation was quantified in the cell-soluble fraction and detected with ELISA. Data are represented as the mean ± SEM of at least three separate experiments. ANOVA model: *p* < 0.0001. * *p* < 0.0001, # *p* < 0.001 vs. control and control + cilastatin; ‡ *p* < 0.0001, † *p* < 0.001, $ *p* < 0.02 vs. same data without cilastatin. Genta, gentamicin; SN, supernatant and a.u., arbitrary units.

The treatment with gentamicin induces dose-dependent apoptosis in RPTECs, as evidenced by cell retraction and rounding and cell detachment (Figure 1A). The quantification of non-adherent cells as an indicator of the presence of cell death by detached cells is shown in Figure 1B. Treatment with cilastatin reduced the number of detached cells and preserved morphology at every gentamicin concentration studied (Figure 1A,B). These data were confirmed by quantification of activated caspase-3 and nucleosomal DNA fragmentation in the cytosol. RPTECs treated with gentamicin showed dose-dependent activation of caspase-3 (Figure 1C,D), as well as an increase in nucleosomes recovered from cytosol (Figure 1E), both of which were significantly prevented by coincubation with cilastatin (Figure 1C–E).

**Figure 2:**
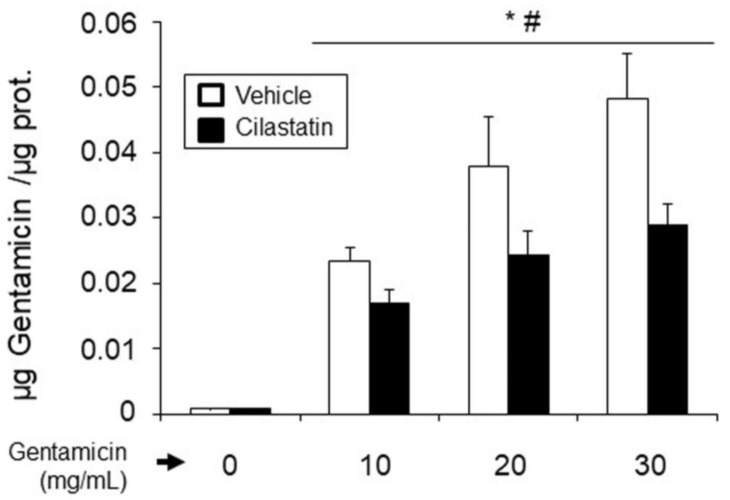
Effects of cilastatin on gentamicin uptake in renal proximal tubular epithelial cells (RPTECs). Gentamicin accumulation was measured in RPTECs-soluble fractions using a fluorescence polarization immunoassay (TDX). RPTECs were treated with gentamicin (10, 20 or 30 mg/mL) in the presence and absence of cilastatin (200 µg/mL) for 24 h. Cilastatin was shown to prevent entry of gentamicin into RPTECs. Values were expressed as the mean ± SEM of the gentamicin concentration (*n* = 4 different experiments). ANOVA model *p* < 0.0001. Factors: cilastatin effect * *p* < 0.05; dose effect # *p* < 0.05.

Analysis of the gentamicin concentration in the soluble fraction of RPTECS showed dose-dependent accumulation that was significantly inhibited by cilastatin (Figure 2).

### 3.2. Cilastatin Ameliorates Body Weight Loss and Renal Function in Rats with Gentamicin-Induced AKI

At the end of the study, gentamicin had caused a marked decrease in body weight gain compared with control rats. Cilastatin significantly prevented weight loss but could not maintain the levels reached in the control group (Table 1).

Gentamicin also significantly increased levels of serum creatinine, BUN and fractional excretion of water and sodium and decreased the GFR in comparison with the control group (Table 2). Cilastatin significantly prevented all these changes and reduced the proteinuria that had previously been increased by gentamicin (Table 2).

Likewise, cilastatin per se did not modify body weight and had no effects on biochemical parameters compared with the control group.

### 3.3. Cilastatin Attenuates Gentamicin-Induced Renal Tubular Damage

**Figure 3:**
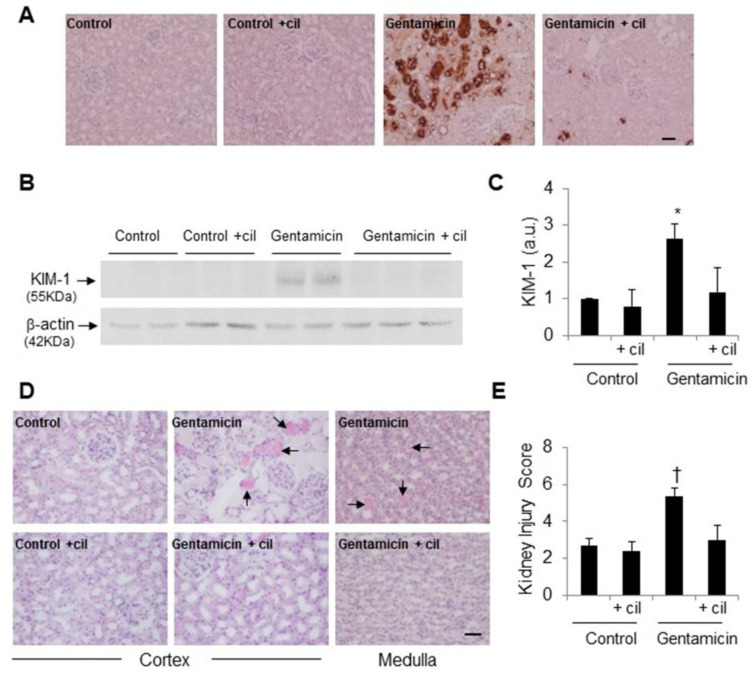
Effects cilastatin on renal histology and injury biomarkers in gentamicin-induced acute renal failure. Animals were treated with 80 mg/kg gentamicin (or its vehicle) and 150 mg/kg cilastatin (or its vehicle) once a day for 8 consecutive days. (**A**) Immunolocalization of kidney injury molecule (KIM)-1 in renal tissue (magnification ×10), bar, 100 µm and (**B**,**C**) representative Western blot of KIM-1 in the renal cortex and densitometric analysis respectively. Results are expressed as the mean ± SEN; *n* = 4–5 animals per group; (**D**) representative images of the renal pathology (hematoxylin–eosin staining, magnification ×20) on day 9 after the first administration of gentamicin. Control groups show normal renal structure; gentamicin-injected kidneys show marked injury with dilation of the tubules, vacuolization, loss of the brush border, sloughing of tubular epithelial cells and intratubular cast formation (arrows). These changes were significantly prevented by treatment with cilastatin; bar, 100 µm; (**E**) semiquantitative renal injury score. Data are expressed as the mean ± SEM; *n* = 6–8 animals per group. * *p* ≤ 0.05, † *p* ≤ 0.005 compared with other groups. Cil, cilastatin and a.u., arbitrary units.

To further confirm the above results, KIM-1 was analyzed in kidney samples. As expected, gentamicin massively increased KIM-1 levels compared with controls, although cilastatin totally reduced its expression (Figure 3A–C).

In regards to its morphology, the kidneys of the control group showed normal histoarchitecture (Figure 3D). The gentamicin group, however, was characterized by extensive structural tubular damage characterized by brush border loss, tubular dilation and necrosis, vacuolization and hyaline casts in renal tubules (Figure 3D). Cotreatment with cilastatin significantly resolved tubular injury compared with the gentamicin group (Figure 3D). These visual results were confirmed by the histological injury score, as shown in Figure 3E.

### 3.4. Cilastatin Reduces Gentamicin-Induced Apoptosis

**Figure 4:**
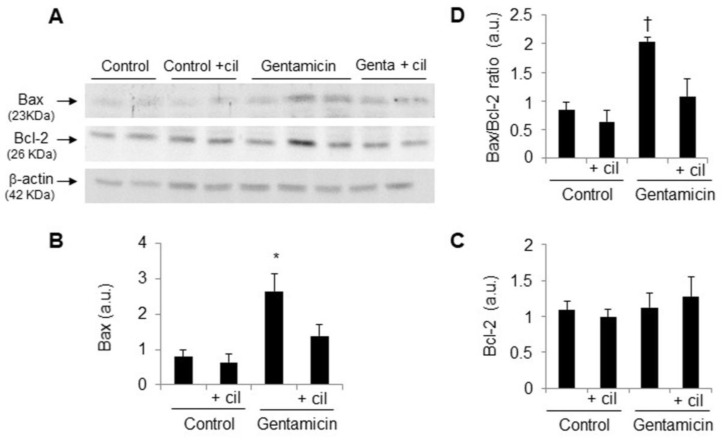
Cilastatin decreases the Bax/Bcl2 ratio in gentamicin-administered rats. Rats were treated with 80 mg/kg gentamicin (or its vehicle) and 150 mg/kg cilastatin (or its vehicle) once a day for 8 consecutive days. (**A**) Representative photomicrographs of the Western blot of Bax and Bcl-2 in the renal cortex and (**B**–**D**) densitometric analysis of Bax, Bcl-2 and Bax/Bcl-2 ratio levels, respectively on Western blots. Cilastatin diminished Bax and Bax/Bcl-2 ratio levels decreasing, therefore, the predisposition to apoptosis. All data are expressed as the mean ± S.E.M.; *n* = 4–5 animals per group. * *p* ≤ 0.02 and † *p* ≤ 0.05 vs. all other groups. Cil, cilastatin; genta, gentamicin and a.u., arbitrary units.

The expression of apoptotic Bax was significantly increased in the gentamicin group compared with the control group, while antiapoptotic Bcl-2 remained unchanged with all of the treatments (Figure 4A–C). Cilastatin prevented the increase in the expression of Bax and reversed the Bax/Bcl-2 ratio, which was previously increased by gentamicin (Figure 4A,B,D), thus indicating that cilastatin decreases the predisposition to apoptosis in the renal tissue.

**Figure 5:**
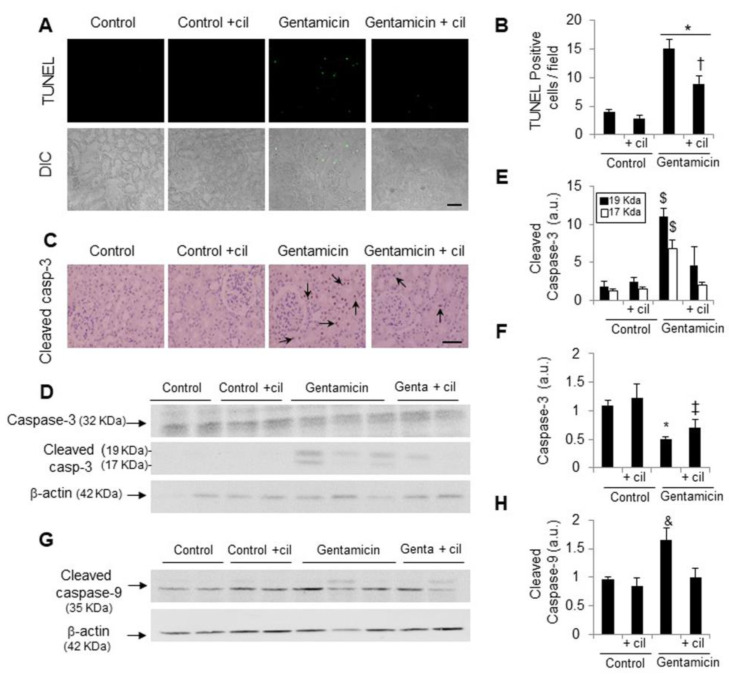
Cilastatin reduces gentamicin-induced caspase-3 expression and apoptosis. Rats were treated with 80 mg/kg gentamicin (or its vehicle) and 150 mg/kg cilastatin (or its vehicle) once a day for 8 consecutive days. (**A**) Photomicrographs of terminal deoxynucleotidyl transferase (TdT)-mediated dUTP nick end labeling (TUNEL) staining in kidney sections. Green fluorescent staining indicates TUNEL-positive nuclei (magnification ×20); (**B**) quantitative analysis of TUNEL-positive cells and (**C**) immunolocalization of active cleaved caspase-3 in kidney sections. Renal tubules are the main site of caspase-3 activation in gentamicin-treated animals (arrows, magnification ×20). Cilastatin significantly decreased the expression of active caspase-3 and TUNEL-positive cells; (**D**) representative photomicrographs of the Western blot of caspase-3 and cleaved caspase-3 in the renal cortex; (**E**,**F**) densitometric analysis of cleaved caspase-3 and caspase-3 respectively on Western blots; (**G**) representative photomicrographs of Western blots of active cleaved caspase-9 in the renal cortex and (**H**) densitometric analysis of active caspase-9 levels on Western blots. Data are expressed as the mean ± SEM.; *n* = 4–5 animals per group for the Western blot and 6–8 animals per group for TUNEL and immunohistochemistry. * *p* ≤ 0.05 vs. control and control + cilastatin groups; † *p* ≤ 0.05 vs. gentamicin group; ‡ *p* ≤0.05 vs. control + cilastatin group; $ *p* ≤ 0.05 and & *p* ≤ 0.02 vs. all other groups. Cil, cilastatin; DIC, differential interference contrast; casp, caspase and a.u., arbitrary units. Bars, 100 µm for each case.

DNA fragmentation in the animal’s kidneys was also measured by the TUNEL technique. As can be seen in Figure 5A,B, the number of TUNEL-positive cells identified by the bright nuclei representing direct cell death was higher than in the control group. Treatment of cilastatin significantly decreased this number protecting the kidneys from gentamicin-induced apoptosis (Figure 5A,B).

In relation to the previous results, cleaved caspase-3 and -9 were increased in the renal cortex after administration of gentamicin compared with untreated kidneys (Figure 5C–H). Cilastatin significantly reduced the high levels of both caspases in gentamicin-treated rats, as assessed by Western blot (Figure 5D,E,G,H) and by immunohistochemistry for caspase-3 (Figure 5C). In contrast, procaspase-3 levels were lower in kidneys treated with gentamicin than in the control groups and recovered with cilastatin (Figure 5D,F).

**Figure 6:**
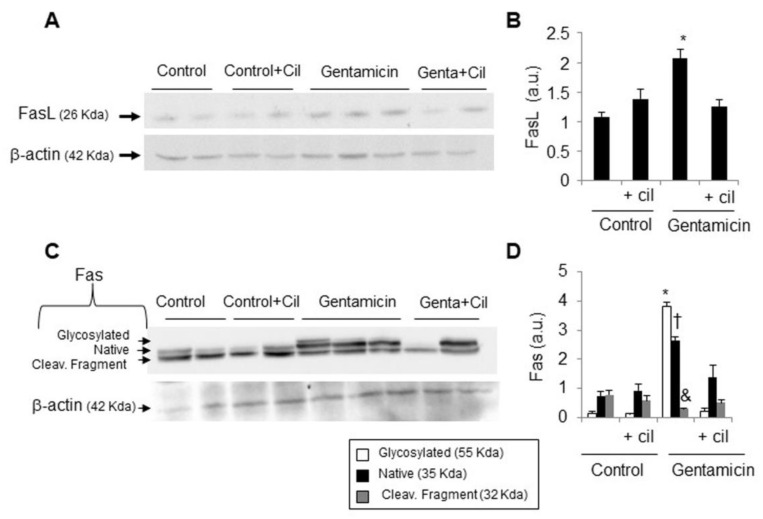
Effect of cilastatin on gentamicin-induced death receptor pathway of apoptosis. Rats were treated with 80 mg/kg gentamicin (or its vehicle) and 150 mg/kg cilastatin (or its vehicle) once a day for 8 consecutive days. (**A**,**C**) Representative photomicrographs of the Western blot of FasL and the Fas receptor, respectively, in the renal cortex and (**B**,**D**) densitometric analysis of FasL and Fas levels on Western blots. Cilastatin diminished gentamicin-induced Fas/FasL complex upregulation. Results are expressed as the mean ± SEM.; *n* = 4–5 animals per group. * *p* ≤ 0.05, † *p* ≤ 0.02 vs. all other groups and & *p* ≤ 0.01 vs. the control group. Cil, cilastatin and a.u., arbitrary units.

On the other hand, the extrinsic pathway of apoptosis was also analyzed. Gentamicin increased renal cortex Fas and FasL levels in comparison with the control group (Figure 6A–D). Cilastatin significantly prevented the expression of both proteins (Figure 6A–D). Compared with the control group, no effects on the above-mentioned variables were found in animals that received only cilastatin.

### 3.5. Cilastatin Reduces Gentamicin-Induced Oxidative Stress

**Figure 7:**
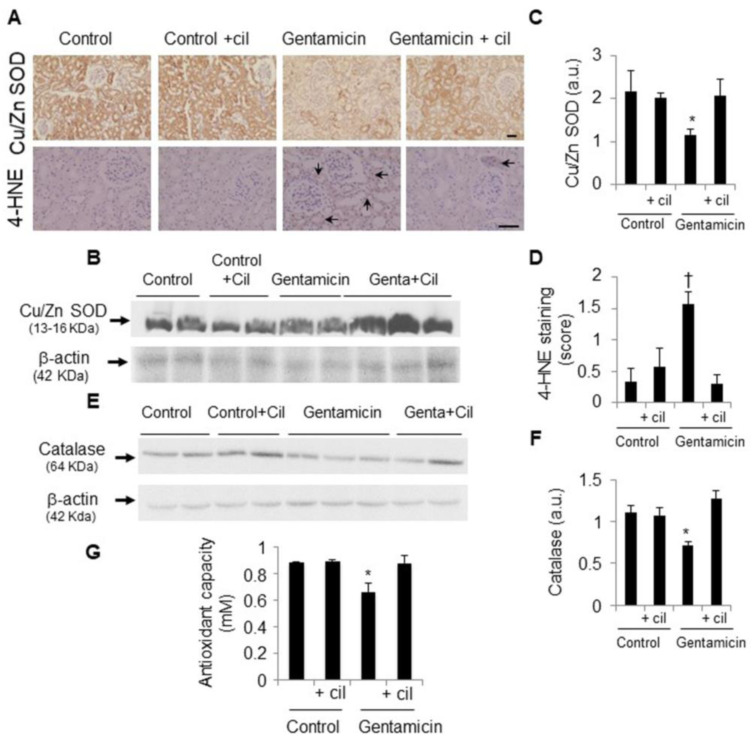
Cilastatin reduces gentamicin-induced oxidative stress. Rats were treated with 80 mg/kg gentamicin (or its vehicle) and 150 mg/kg cilastatin (or its vehicle) once a day for 8 consecutive days. (**A**) Immunolocalization of Cu/Zn superoxide dismutase (SOD) and 4-hydroxy-2-nonenal (4-HNE) in the kidney sections. Note increased lipid peroxidation (tubular staining) in gentamicin-injected rats (arrows) and decreased Cu/Zn SOD expression in the same animals compared with gentamicin + cilastatin and control rats (magnification ×20), bars, 100 µm; (**B**,**C**) representative photomicrographs of the Western blot of Cu/Zn SOD in the renal cortex and densitometric analysis, respectively, and (**D**) quantification of 4-HNE immunostaining in the kidney sections. Cilastatin decreases gentamicin-induced lipid peroxidation; (**E**,**F**) representative photomicrographs of the Western blot of catalase in kidney samples and densitometric analysis, respectively, and (**G**) quantification of the antioxidant capacity in urine using specific ELISA kits. Data are expressed as the mean ± SEM.; *n* = 4–5 animals per group for the Western blot and 6–8 animals per group for immunohistochemistry and antioxidant capacity. * *p* ≤ 0.05 and † *p* ≤ 0.005 compared with all other groups. Cil, cilastatin and a.u., arbitrary units.

Administration of gentamicin increased OS and decreased total antioxidant defense capacity in comparison with the control group (Figure 7). 4-HNE, an oxidative damage biomarker, was increased in gentamicin-treated rats (Figure 7A,D). In contrast, two of the main antioxidant proteins, such as Cu/Zn SOD and catalase, were diminished in the renal tissue (Figure 7A–C,E,F), leading to decreased antioxidant capacity at the systemic level (Figure 7G). Treatment with cilastatin totally restored the expression of both proteins in the kidney and led to systemic recovery of antioxidant capacity levels, while lipid peroxidation was diminished (Figure 7).

**Figure 8:**
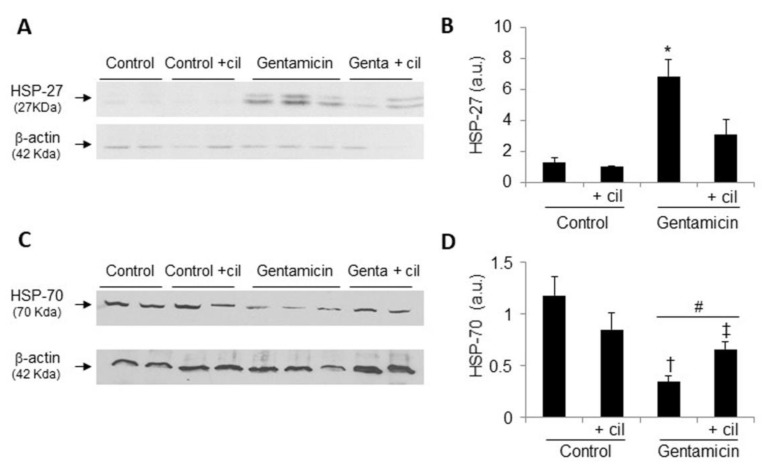
Effects of gentamicin and cilastatin on the heat shock protein (HSPs) expression. Rats were treated with 80 mg/kg gentamicin (or its vehicle) and 150 mg/kg cilastatin (or its vehicle) once a day for 8 consecutive days. (**A**,**C**) Representative photomicrographs of the Western blot of HSP-27 and HSP-70, respectively, and (**B**,**D**) densitometric analysis of HSP-27 and HSP-70 levels on Western blots, respectively. Cilastatin diminished HSP-27 and increased HSP-70 were previously modified both by gentamicin. Data are expressed as the mean ± SEM.; *n* = 4–5 animals per group. * *p* ≤ 0.005 vs. all other groups; # *p* ≤ 0.05 vs. control group; † *p* ≤ 0.05 vs. control + cilastatin group and ‡ *p* ≤0.05 vs. gentamicin group. Cil, cilastatin; genta, gentamicin and a.u., arbitrary units.

In addition, it is known that HSPs protect cells from OS and cell death, thus increasing antioxidant defenses. In fact, the expression of HSP-27 in the renal cortex was increased after treatment with gentamicin compared with controls, although, in contrast, HSP-70 was decreased. Cilastatin significantly recovered the levels of both proteins (Figure 8). Cilastatin by itself had no effect on any of these variables in comparison with the control group.

### 3.6. Cilastatin Reduces Gentamicin-Induced Renal Inflammation

**Figure 9:**
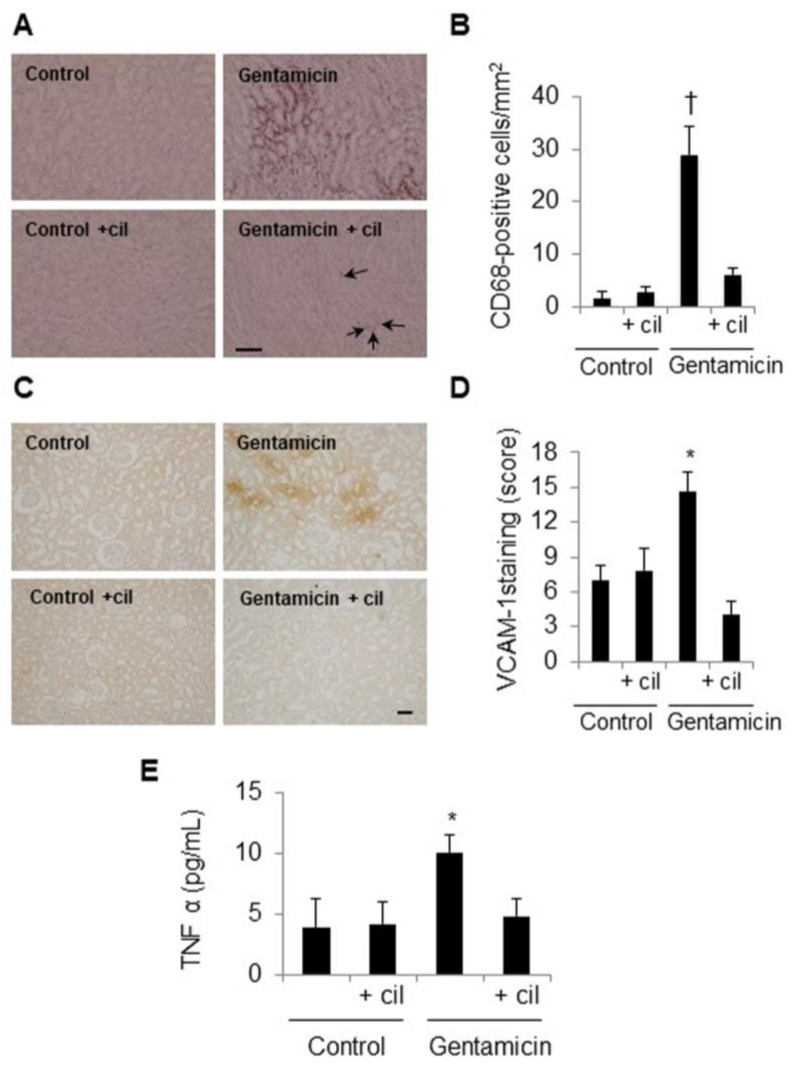
Cilastatin decreases gentamicin-induced inflammation. Rats were treated with 80 mg/kg gentamicin (or its vehicle) and 150 mg/kg cilastatin (or its vehicle) once a day for 8 consecutive days. (**A**) Localization of monocyte/macrophage (CD68) in the renal sections of animals. Note: increased staining in gentamicin-injected rats compared with gentamicin + cilastatin (little staining, arrows) and control rats (magnification × 10); bar, 100 µm. (**B**) Quantification of CD68 immunostaining in renal cells. (**C**) Immunolocalization of vascular cell adhesion molecule 1 (VCAM-1) in the kidney sections of the different animal groups. Note: increased staining in gentamicin-injected rats compared with gentamicin + cilastatin and control rats (magnification ×10), bar, 100 µm. (**D**) Quantification of VCAM-1 immunostaining in the kidney sections. (**E**) Serum TNFα levels in the groups of rats studied. Administration of gentamicin increases systemic TNFα concentration. This change was significantly prevented with the cilastatin treatment. All results are expressed as the mean ± SEM; *n* = 6–8 animals per group. * *p* ≤ 0.05 and † *p* ≤ 0.005 compared with all other groups. Cil, cilastatin and a.u., arbitrary units.

The treatment with gentamicin also increased inflammation in the kidneys. Specifically, these kidneys had greater numbers of CD68-positive cells (representing recruitment of monocytes/macrophages) than the controls (Figure 9A,B). As expected, expression of VCAM-1, which promotes recruitment of monocytes/macrophages, was also increased (Figure 9C,D). Treatment with cilastatin completely normalized adhesion molecule levels and abolished the increase in infiltrating cells protecting the kidneys (Figure 9A–D). To confirm these results, TNFα levels were evaluated in serum. Cilastatin decreased the TNFα levels that had previously been increased by gentamicin (Figure 9E), thus preventing cell infiltration and exacerbation of kidney damage.

**Figure 10:**
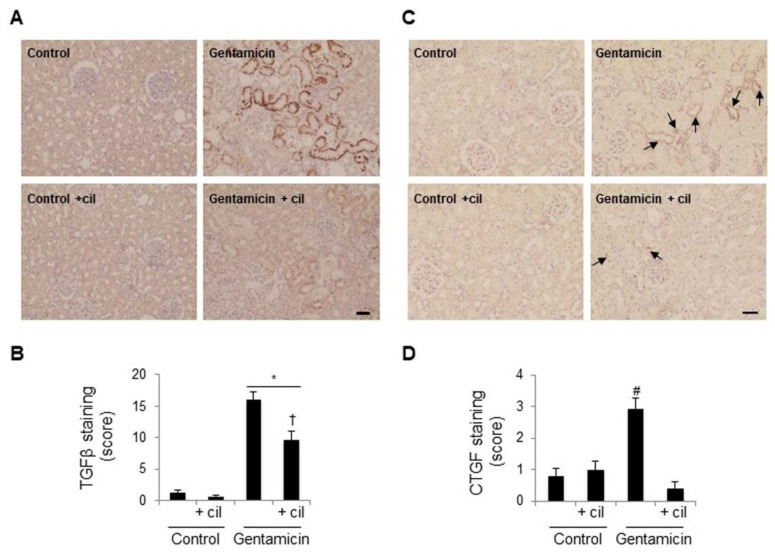
Cilastatin reduces gentamicin-induced increased expression of TGFβ and CTGF. Rats were treated with 80 mg/kg gentamicin (or its vehicle) and 150 mg/kg cilastatin (or its vehicle) once a day for 8 consecutive days. (**A**,**C**) Immunolocalization of the transforming growth factor beta (TGFβ) and connective tissue growth factor (CTGF), respectively, in the kidney sections of animals. Note: increased staining in gentamicin-injected rats in both factors (arrows for CTGF staining) compared with gentamicin + cilastatin (less staining, arrows for CTGF staining) and control rats (magnification ×10); bars, 100 µm. (**B**,**D**) Quantification of TGFβ and CTGF immunostaining respectively in kidney sections. Results are expressed as the mean ± SEM; *n* = 6–8 animals per group. * *p* ≤ 0.001 vs. control and control+cilastatin; † *p* ≤ 0.05 vs. gentamicin group and # *p* ≤ 0.05 compared with all other groups. Cil, cilastatin and a.u., arbitrary units.

TGFβ and CTGF are two of the main profibrotic and proinflammatory factors involved in the exacerbation and amplification of inflammatory damage in the kidney, due to their regulatory and activating inflammatory capacity. Immunohistochemical studies showed elevated levels of both growth factors in the kidneys of gentamicin-treated animals compared to the control group (Figure 10). Consistent with the above results, treatment with cilastatin significantly decreased the presence of TGFβ and CTGF protecting the kidneys from inflammatory damage (Figure 10). Again, treatment with cilastatin had no effect on all of these variables in comparison with the control group.

### 3.7. Effects of Cilastatin on Intracellular Gentamicin Accumulation and Megalin Expression

**Figure 11:**
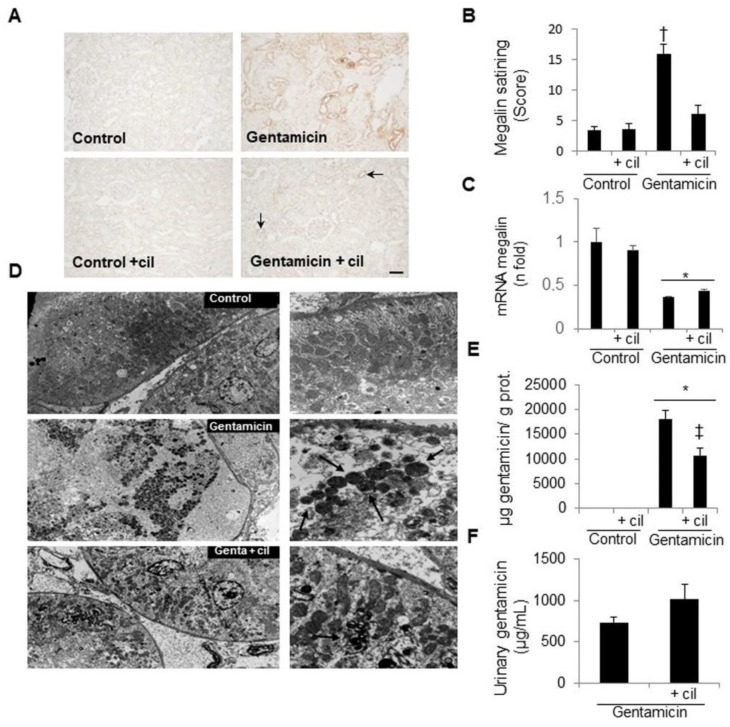
Effects of cilastatin on gentamicin uptake, accumulation and excretion and on the expression of its transporter, megalin in gentamicin-injected rats. Rats were treated with 80 mg/kg gentamicin (or its vehicle) and 150 mg/kg cilastatin (or its vehicle) once a day for 8 consecutive days. (**A**) Immunolocalization of megalin in the kidney sections of animals. Note increased staining in gentamicin-injected rats in comparison with gentamicin + cilastatin (little staining, arrows) and control rats (magnification ×10); bar, 100 µm. (**B**) Quantification of megalin immunostaining in the renal cells. (**C**) Renal mRNA expression of megalin. Cilastatin significantly decreased the protein megalin levels that were previously increased by gentamicin administration. In contrast, cilastatin increased partially (although not significantly) the megalin mRNA levels that were previously decreased by gentamicin. (**D**) Representative images of electron microscopy of the renal tissue. Normal kidneys do not show the presence of myeloid bodies, but these can be clearly observed in rats receiving gentamicin alone (arrows). Cilastatin coadministration decreases its number (arrows). (**E**) Gentamicin concentration in the kidneys of the animals 9 days after gentamicin administration. Cilastatin reduces gentamicin uptake and accumulation. (**F**) Urinary excretion of gentamicin. The results show an increase of gentamicin in the urine of animals cotreated with gentamicin and cilastatin. Data are expressed as the mean ± SEM.; *n* = 6–8 animals per group. * *p* ≤ 0.001 vs. control and control + cilastatin groups; † *p* ≤ 0.01 compared with all other groups and ‡ *p* ≤ 0.01 vs. gentamicin group. Cil, cilastatin; genta, gentamicin and prot., protein.

It is known that alterations in megalin expression indicate changes in the uptake of the antibiotic. Gentamicin overexpressed megalin protein levels, but, in contrast, it decreased megalin mRNA (Figure 11A–C). Treatment with cilastatin significantly diminished megalin protein levels in kidneys treated with gentamicin, thus leading to a decrease in the internalization of gentamicin into the tubular cells, although it had little or no effect on the megalin gene (Figure 11E). As expected, this result was similar to that found in vitro (Figure 2). Consistent with these findings, urinary excretion of gentamicin was higher in the group treated with cilastatin than in that treated with gentamicin alone, although the difference did not reach statistical significance (Figure 11F).

Moreover, electron microscopy images of gentamicin-treated rats showed many deposits of the antibiotic in myeloid bodies throughout the cell cytoplasm compared with control animals, where no myeloid bodies were observed. Downregulation of megalin and the subsequent reduced accumulation of gentamicin in the cells of rats cotreated with cilastatin caused a marked loss in the presence of myeloid bodies (Figure 11D).

### 3.8. Cilastatin Has No Effect on the Bactericidal Actions of Gentamicin

The effect of cilastatin on gentamicin-induced bacteria killing in isolates of *Staphylococcus aureus* and *Escherichia coli* strains was analyzed.

The same values or values that varied within a ±1 log2 dilution (Table 3) were obtained when measuring MICs and MBCs of gentamicin for each bacteria strain in the presence or absence of cilastatin (200 µg/mL), therefore showing that cilastatin does not alter the antibacterial efficiency of gentamicin against any of the strains tested in this assay.

The same values or values that varied within a ±1 log2 dilution (Table 3) were obtained when measuring MICs and MBCs of gentamicin for each bacteria strain in the presence or absence of cilastatin, therefore showing that cilastatin does not alter the antibacterial efficiency of gentamicin against any of the strains tested in this assay.

## 4. Discussion

Gentamicin is a widely used aminoglycoside antibiotic that has proven efficiency against life-threatening Gram-negative bacterial infections. However, it causes nephrotoxicity, which limits its use considerably, lengthens the hospital stay and worsens the patient’s prognosis [2,21]. In this study, we demonstrated both in vitro and in vivo that administration of cilastatin significantly reduces gentamicin-induced nephrotoxicity without affecting its therapeutic value.

Apoptosis is a central event in gentamicin-induced cytotoxicity, and it has been observed both in vivo and ex vivo [2,22,23,24,25,26,27]. Confirming this evidence, activated caspase-3 was increased by gentamicin in a dose-dependent manner in the RPTECs, leading to DNA degradation and increased cell loss and cell death. Previous studies conducted by our group have shown that cilastatin selectively protects the RPTECS against adverse effects of chemotherapy agents [14], immunosuppressants [13] and analgesics [18], thus reducing apoptosis. The 200 μg/mL dose was effective in those cases and has also proven efficiency against the adverse effects of gentamicin. In fact, cilastatin significantly decreased caspase-3 activation at all the concentrations of gentamicin used and, therefore, decreased DNA degradation and condensation, as well as cell loss.

All these results were also corroborated in vivo. Cilastatin reduced the onset of apoptosis in the kidneys through interference with the upregulation of Bax, cleaved caspase-9 and -3, FasL and Fas, all of which were increased by gentamicin.

Administration of gentamicin to rats caused morphological kidney damage and increased creatinine, BUN, KIM-1 and proteinuria among others. It also decreased the GFR and body weight. These effects are similar to those found by other authors using similar models [3,28,29,30,31]. All of these parameters were recovered significantly (partially or totally) with the administration of cilastatin, which led to an improvement at the morphological and biochemical level.

ROS and inflammation are key processes in gentamicin-induced nephrotoxicity in their role as amplifiers of AKI [2,8,11,24,27]. Cilastatin reversibly binds to DHP-I to inhibit renal degradation of imipenem. Therefore, it is not an antioxidant or an anti-inflammatory drug. However, cilastatin protects the kidneys from the oxidative and inflammatory toxic damage induced by gentamicin. Indeed, gentamicin increased oxidation, as evidenced by the increase in lipid peroxidation, probably as a result of the decrease in the total antioxidant capacity (catalase and Cu/Zn SOD). Cilastatin restored this situation in a similar way to that found by other authors using antioxidants as nephroprotective agents [24,28,32]. Indeed, there is a direct relationship between the presence of stress and activation/inhibition of HSPs [16] and in several models of nephrotoxicity, HSP-27 is overexpressed owing to its antioxidant and antiapoptotic role [33]. Likewise, HSP-70 has a reduced function when gentamicin specifically binds to it, which has been linked to increased renal toxicity [34,35]. Our results are consistent with these observations, showing increased HSP-27 and decreased HSP-70 in the kidneys after treatment with gentamicin. Cilastatin restored the levels of both HSPs, thus helping to mitigate the effect of gentamicin, as other authors have shown [35].

Our findings corroborate the inflammatory response induced by gentamicin, showing greater activation of TNFα followed by an increase in VCAM-1 and infiltration of monocyte/macrophage and profibrotic and proinflammatory factors, which act together to amplify damage. Cilastatin again abolished all signs of gentamicin-induced inflammation.

It is well known that gentamicin-induced AKI occurs mainly after accumulation of the drug inside the cells [1,2,5,7,8] and that megalin plays a critical role in binding and the uptake of gentamicin in RPTECs [5,7,35,36]. In fact, it has been proposed as the main route for the entry and accumulation of gentamicin and therefore the main target in the prevention of gentamicin-induced nephrotoxicity [1,2,5,7,35,36,37]. Very recently, Hori et al. concluded that the megalin blockade with cilastatin efficiently suppresses the nephrotoxicity induced by antibiotics (gentamicin, colistin and vancomycin) and chemotherapy agents (cisplatin), although they could not determine the Kd value for binding gentamicin, cisplatin or cilastatin to megalin [36]. In our results, phospholipidosis causes both megalin sequestration and accumulation to a degree that its synthesis is reduced, decreasing the specific mRNA. Taking megalin as an indirect indicator of the entry of gentamicin, cilastatin completely decreased the megalin levels that had been previously increased by gentamicin in animal kidneys; likewise, urinary excretion of gentamicin was increased when cilastatin was present. Similar results were found by Cardenas-Gonzalez et al. when coadministering fluoride with gentamicin [35]. Therefore, our results showed a partial decrease in the entry and accumulation of gentamicin both in vitro (for all doses used) and in vivo when cilastatin was coadministered, thus explaining only partly the decrease in AKI induced by gentamicin. In fact, in animals treated with gentamicin and cilastatin, lipidosis can still be observed at a lower level (lower presence of myeloid bodies) and even though mRNA levels of megalin are partially recovered, these levels are still below the ones of those of the control animals. As claimed by Hori et al., cilastatin inhibition from the endocytosis process triggered by ligand–megalin binding may explain most changes in gentamicin intracellular concentrations [36] or other drugs [14,15,18]. However, in our results, the small reduction of gentamicin in intracellular concentration induced by cilastatin does not fully explain the observed effects on apoptosis and it does not explain the overwhelming morphological and biochemical protection.

Therefore, our findings indicate that other mechanisms of action could also be possible. In fact, we reported that binding of cilastatin to CLR-bound DHP-I inhibits internalization and then transport and signaling of the brush border CLR in proximal tubules providing protection [13,14,15,16,17,18]. DHP-I is located primarily in the brush border of RPTECs and is anchored specifically to CLR [14]. The same apical CLR host cholera toxin receptors (ganglioside GM1) [38], megalin [39] and Fas expressed during drug-induced renal toxicity [40]. It has been demonstrated that binding of a ligand to its receptor in the CLR accelerates the internalization of the rafts [13,18]. In previous studies, we showed that RPTECs were invaded through internalization of labeled cholera toxin to a perinuclear position, while the same cells coincubated with cilastatin show the fluorescent label attached to the membrane. Interference with internalization of the cholera toxin receptor strongly suggests a CLR-dependent endocytic pathway [18] and, therefore, the cycling processes associated with CLR [13,14,16,18].

Binding of FasL causes translocation and aggregation of Fas into CLR clusters to trigger the extrinsic pathway of apoptosis [40,41,42]. When RPTECs were treated with cisplatin in the presence of cilastatin, CLR internalization was blocked by the cilastatin/DHP-I complex, thereby inhibiting the extension phase of renal cell damage [14,16]. The same results were obtained with gentamicin in this study. Cilastatin does not prevent the initial damage produced by gentamicin, but it does prevent its amplification by avoiding the internalization of the Fas/FasL complex and the initiation of the extrinsic pathway of apoptosis, with the consequent reduction of oxidative and inflammatory mechanisms. In accordance with our results, cholesterol-depleting agents, which disrupt CLR, block the internalization of cholera toxin and FasL-induced apoptosis and prevent Fas clustering in response to stimulation by an antibody and other substances and processes in cells [13,43]. We previously reported on the modification of the physical properties of membranes resulting from the interaction between cilastatin and DHP-I-bound CLR [13]. As stated above, megalin is located coincidentally in the apical CLR [39]. Therefore, in these membrane microdomains, megalin is located within the same location as other entities such as cholera toxin receptor, Fas and DHP-I. Binding of cilastatin to DHP-I—gentamicin, cisplatin, FasL and the cholera toxin are not substrates for the catalytic activity of renal DHP-I—blocks the extrinsic pathway of the apoptosis and the uptake of the cholera toxin and many nephrotoxic antibiotics inside the cells, because CLR cycling is interrupted, probably by steric hindrance or conformational changes [13,14,15,16,17,18]. Therefore, we believe that the direct blockade of megalin with cilastatin is not the only explanation for total protection against gentamicin (and other drugs) and that blockage of CLR internalization and transport would produce identical results. This will cause a disruption or alteration of megalin–gentamicin endocytosis (reducing gentamicin entry) and Fas/FasL endocytosis (reducing cell death and amplification of damage). This observation could also explain the protective effects of cilastatin against other drugs whose mode of action, to our knowledge, is not affected by the megalin function, e.g., radiocontrast agents. In fact, Lau et al. recently published the role of DHP-I in the uptake of radiocontrast agents in induced AKI and the protection afforded by cilastatin against oliguric kidney injury [44].

## 5. Conclusions

**Figure 12:**
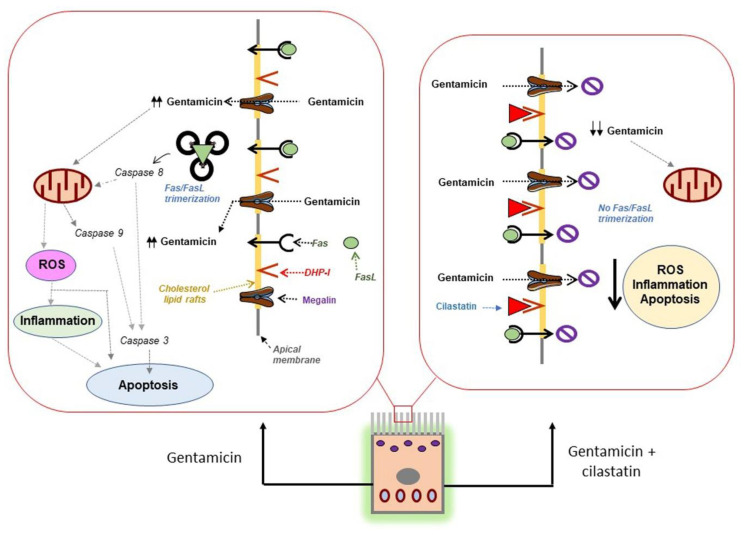
Summary of the postulated protective mechanism of cilastatin against gentamicin-induced AKI. Megalin, an endocytic receptor located in the cholesterol lipid rafts on the brush border apical side of renal proximal tubular epithelial cells, is the main route for the entry and accumulation of gentamicin. On the left panel, intracellular gentamicin promotes direct tubular damage affecting the mitochondria and increased Fas expression that leads (after its clustering with FasL) to apoptosis and cell death and finally to oxidative stress and inflammation, which exacerbates and amplifies the kidney injury. On the right panel, cilastatin binding to the brush border membrane dehydropeptidase-I (DHP-I) in cholesterol lipids rafts causes a significant reduction in gentamicin uptake and cancels the initial step of extrinsic pathway of gentamicin-induced proximal cell apoptosis, reducing caspase 8, 3 and 9 activations, and oxidative and proinflammatory signaling pathways, thereby protecting tubular cells. ROS, reactive oxygen species.

In conclusion, our results show for the first time that cilastatin reduced renal toxicity in gentamicin-induced AKI both in vitro and in vivo. The mechanism of the beneficial effect could be partially attributed to a decrease in accumulation of the drug by the cells and mainly by the cancellation of key steps in the damage amplification owing to the blockade of CLR internalization (Figure 12). Therefore, cilastatin may represent a new therapeutic option for the preservation of the renal function in gentamicin-treated patients without compromising the efficacy of bactericidal therapy.

## 6. Patents

The following patents are in part due to the work done in this manuscript:

“Use of cilastatin to reduce nephrotoxicity of various compounds”. Patent numbers: EP 2143429 B1; US 9,216,185 B2; US 9,522,128 B2 and US-9757349-B2. The holder of the rights is Fundación para la Investigación Biomédica del Hospital Gregorio Marañón (FIBHGM).

## Figures and Tables

**Table 1:** The effect of cilastatin on body weight in gentamicin-treated rats.

Groups	Initial Weight (g)	Final Weight (g)	Δ Weight (g)
Control	296 ± 12	312 ± 10	16 ± 3
Control + Cil	287 ± 8	299 ± 8	12 ± 2
Gentamicin	268 ± 9	265 ± 9	−3 ± 2 ^a^
Gentamicin + Cil	283 ± 9	288 ± 5	5 ± 3 ^b, c^

Table shows the body weight in the different study groups at the beginning and end of the study. Animals were treated with 80 mg/kg gentamicin (or its vehicle) and 150 mg/kg cilastatin (or its vehicle) once a day for 8 consecutive days. Results are expressed as the mean ± SEM: *n* = 7–8 animals per group. ^a^ *p* < 0.01 vs. control and control + cilastatin; ^b^ *p* < 0.05 vs. control and ^c^ *p* < 0.05 vs. gentamicin. Cil, cilastatin.

**Table 2:** The effects of cilastatin on gentamicin-induced nephrotoxicity in rats.

Groups	S_Creat_ (mg/dL)	BUN (mg/dL)	GFR (mL/min/100 g)	U_Prot_ (mg/24 h)	FE_Na_^+^	FE_H2O_
Control	0.24 ± 0.03	30.00 ± 3.48	0.91 ± 0.08	9.12 ± 0.57	0.27 ± 0.02	0.24 ± 0.03
Control + Cil	0.27 ± 0.02	30.00 ± 1.32	0.72 ± 0.05	8.65 ± 0.66	0.27 ± 0.03	0.28 ± 0.04
Gentamicin	0.82 ± 0.12 ^a^	55.75 ± 7.10 ^e^	0.26 ± 0.04 ^b^	26.54 ± 4.06 ^f^	1.22 ± 0.17 ^a^	3.27 ± 0.51 ^g^
Gentamicin + Cil	0.50 ± 0.07 ^a,c^	38.67 ± 3.61	0.46 ± 0.09 ^b,d^	16.67 ± 2.09	0.76 ± 0.10 ^a,c^	1.02 ± 0.22

Rats were treated with 80 mg/kg gentamicin (or its vehicle) and 150 mg/kg cilastatin (or its vehicle) once a day for 8 consecutive days. Results are expressed as the mean ± SEM; ^a^ *p* < 0.01, ^b^ *p* < 0.05 vs. control and control + cilastatin; ^c^ *p* < 0.01, ^d^ *p* < 0.05 vs. gentamicin; ^e^ *p* < 0.02, ^f^ *p* < 0.01 and ^g^ *p* < 0.001 vs. all other groups. Cil, cilastatin; SCreat, serum creatinine; BUN, blood urea nitrogen; GFR, glomerular filtration rate; UProt, urinary protein and FE, fractional excretion.

**Table 3:** In vitro activity of gentamicin in the presence or absence of cilastatin against clinical isolates of *Staphylococcus aureus* and *Escherichia coli*.

	Strain 1		Strain 2		Strain 3		Strain 4	
***Staphylococcus aureus***	Cil	Vehicle	Cil	Vehicle	Cil	Vehicle	Cil	Vehicle
MIC	0.5	0.5	0.5	0.25	0.5	0.5	0.5	0.5
MBC	0.5	0.5	0.5	0.5	1	1	0.5	0.5
***Escherichia coli***	Cil	Vehicle	Cil	Vehicle	Cil	Vehicle	Cil	Vehicle
MIC	1	0.5	1	0.5	0.5	0.5	0.5	1
MBC	1	0.5	1	0.5	0.5	0.5	0.5	1

The table shows the effect of cilastatin (200 μg/mL) against the bactericidal and inhibitory effect of gentamicin (0.0625–64 μg/mL) in isolated clinical bacteria. *Staphylococcus aureus*: strains numbers 1 and 4, methicillin-resistant and strain numbers 2 and 3, methicillin-susceptible. MIC, minimum inhibitory concentration; MBC, minimum bactericidal concentration; vehicle, cation-adjusted Mueller–Hinton broth and cil, cilastatin.
